# Bis{(1-methyl­imidazol-2-ylmeth­yl)[2-(2-pyridyl)eth­yl]amine-κ^3^ *N*,*N*′,*N*′′}zinc(II) bis­(hexa­fluoridophosphate)

**DOI:** 10.1107/S1600536809044419

**Published:** 2009-10-31

**Authors:** Ai-Zhi Wu, Seik Weng Ng

**Affiliations:** a School of Chinese Materia Medica, Guangzhou University of Chinese Medicine, Guangzhou 510006, People’s Republic of China; b Department of Chemistry, University of Malaya, 50603 Kuala Lumpur, Malaysia

## Abstract

Two tridentate *N*-heterocyclic ligands chelate the Zn^II^ atom in the title compound, [Zn(C_12_H_16_N_4_)_2_](PF_6_)_2_, conferring a *fac*-octa­hedral geometry. The Zn^II^ atom lies on a center of inversion. The cation is linked to the anion by an N—H⋯F hydrogen bond.

## Related literature

No crystal structure studies of metal complexes with the *N*-heterocyclic ligand have been reported. For the synthesis of the ligand, see: Greatti *et al.* (2008 ▶).
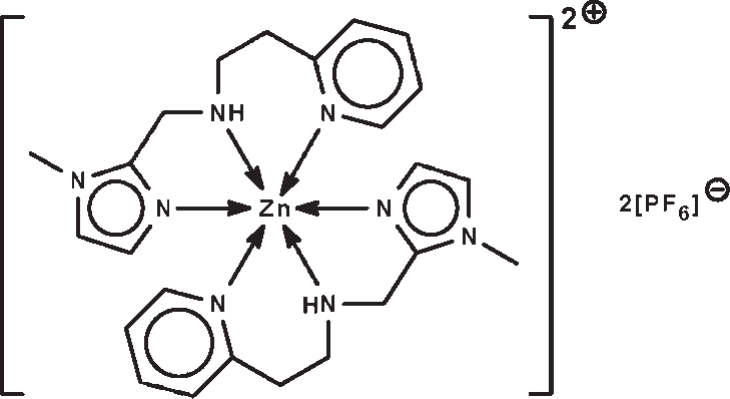


## Experimental

### 

#### Crystal data

[Zn(C_12_H_16_N_4_)_2_](PF_6_)_2_
*M* *_r_* = 787.89
Orthorhombic, 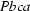

*a* = 13.3147 (5) Å
*b* = 11.8147 (4) Å
*c* = 20.3359 (6) Å
*V* = 3199.0 (2) Å^3^
*Z* = 4
Mo *K*α radiation
μ = 0.97 mm^−1^
*T* = 100 K
0.40 × 0.38 × 0.35 mm

#### Data collection

Bruker APEXII diffractometer
Absorption correction: multi-scan (*SADABS*; Sheldrick, 1996 ▶) *T*_min_ = 0.698, *T*_max_ = 0.728
18287 measured reflections
3655 independent reflections
2740 reflections with *I* > 2σ(*I*)
*R*_int_ = 0.048

#### Refinement

*R*[*F* ^2^ > 2σ(*F* ^2^)] = 0.052
*wR*(*F* ^2^) = 0.147
*S* = 1.04
3655 reflections
219 parameters
1 restraint
H atoms treated by a mixture of independent and constrained refinement
Δρ_max_ = 1.49 e Å^−3^
Δρ_min_ = −0.71 e Å^−3^

### 

Data collection: *APEX2* (Bruker, 2005 ▶); cell refinement: *SAINT* (Bruker, 2005 ▶); data reduction: *SAINT*; program(s) used to solve structure: *SHELXS97* (Sheldrick, 2008 ▶); program(s) used to refine structure: *SHELXL97* (Sheldrick, 2008 ▶); molecular graphics: *X-SEED* (Barbour, 2001 ▶); software used to prepare material for publication: *publCIF* (Westrip, 2009 ▶).

## Supplementary Material

Crystal structure: contains datablocks global, I. DOI: 10.1107/S1600536809044419/xu2647sup1.cif

Structure factors: contains datablocks I. DOI: 10.1107/S1600536809044419/xu2647Isup2.hkl

Additional supplementary materials:  crystallographic information; 3D view; checkCIF report

## Figures and Tables

**Table 1:** Selected bond lengths (Å)

Zn1—N1	2.188 (3)
Zn1—N2	2.095 (3)
Zn1—N4	2.298 (3)

**Table 2:** Hydrogen-bond geometry (Å, °)

*D*—H⋯*A*	*D*—H	H⋯*A*	*D*⋯*A*	*D*—H⋯*A*
N1—H1⋯F1	0.88 (1)	2.21 (1)	3.083 (4)	179 (3)

**Fig. 1.**
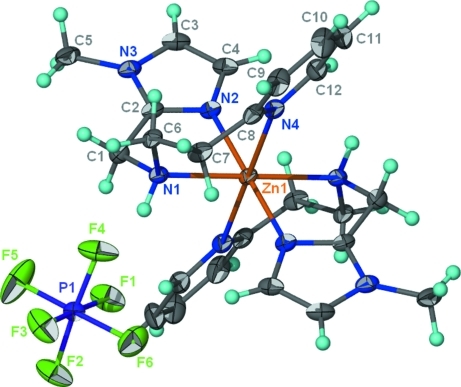
Thermal ellipsoid plot (Barbour, 2001) of [Zn(C12H16N4)2] 2[PF6] at the 70% probability level; hydrogen atoms are drawn as spheres of arbitrary radius.

## supplementary crystallographic information

### Experimental

(1-Methylimidazol-2-ylmethyl)(pyridin-2-ylethyl)amine was synthesized according to a literature method (Greatti *et al.*, 2008).

The ligand (1 mmol, 0.26 g) dissolved in methanol (5 ml) was reacted with zinc hexafluorophosphat (1 mmol, 0.36 g) dissolved in water (5 ml). The mixture was filtered and the solution set aside for the growth of colorless block-shaped crystals that formed after several days in 60% yield. CH&N elemental analysis. Found: C 36.24, H 4.07, N 13.36%; calculated: C 36.55, H 4.06, N 14.22%.

### Refinement

Carbon-bound H-atoms were placed in calculated positions (C—H 0.95 to 0.99 Å) and were included in the refinement in the riding model approximation, with *U*(H) set to 1.2 to 1.5*U*(C).

The imino H-atom was located in a difference Fourier map, and was refined with a distance restraint of N–H 0.88±0.01 Å; its temperature factor was freely refined.

The final difference Fourier map had a peak in the vicinity of Zn1 but was otherwise featureless.

### Crystal data

**Table:**

[Zn(C_12_H_16_N_4_)_2_](PF_6_)_2_	*F*(000) = 1600
*M**_r_* = 787.89	*D*_x_ = 1.636 Mg m^−^^3^
Orthorhombic, *P**b**c**a*	Mo *K*α radiation, λ = 0.71073 Å
Hall symbol: -P 2ac 2ab	Cell parameters from 4790 reflections
*a* = 13.3147 (5) Å	θ = 2.5–28.2°
*b* = 11.8147 (4) Å	µ = 0.97 mm^−^^1^
*c* = 20.3359 (6) Å	*T* = 100 K
*V* = 3199.0 (2) Å^3^	Block, colorless
*Z* = 4	0.40 × 0.38 × 0.35 mm

### Data collection

**Table:**

Bruker APEXII diffractometer	3655 independent reflections
Radiation source: fine-focus sealed tube	2740 reflections with *I* > 2σ(*I*)
graphite	*R*_int_ = 0.048
ω scans	θ_max_ = 27.5°, θ_min_ = 2.0°
Absorption correction: multi-scan (*SADABS*; Sheldrick, 1996)	*h* = −10→17
*T*_min_ = 0.698, *T*_max_ = 0.728	*k* = −13→15
18287 measured reflections	*l* = −22→26

### Refinement

**Table:**

Refinement on *F*^2^	Primary atom site location: structure-invariant direct methods
Least-squares matrix: full	Secondary atom site location: difference Fourier map
*R*[*F*^2^ > 2σ(*F*^2^)] = 0.052	Hydrogen site location: inferred from neighbouring sites
*wR*(*F*^2^) = 0.147	H atoms treated by a mixture of independent and constrained refinement
*S* = 1.04	*w* = 1/[σ^2^(*F*_o_^2^) + (0.0755*P*)^2^ + 5.7073*P*] where *P* = (*F*_o_^2^ + 2*F*_c_^2^)/3
3655 reflections	(Δ/σ)_max_ = 0.001
219 parameters	Δρ_max_ = 1.49 e Å^−^^3^
1 restraint	Δρ_min_ = −0.71 e Å^−^^3^

### Fractional atomic coordinates and isotropic or equivalent isotropic displacement parameters (Å^2^)

**Table:**

	*x*	*y*	*z*	*U*_iso_*/*U*_eq_	
Zn1	0.5000	0.5000	0.5000	0.02098 (16)	
P1	0.38657 (6)	0.83958 (7)	0.68434 (4)	0.0251 (2)	
F1	0.47058 (17)	0.7507 (2)	0.66130 (14)	0.0524 (7)	
F2	0.47025 (19)	0.9039 (2)	0.72513 (13)	0.0563 (7)	
F3	0.30477 (18)	0.9306 (2)	0.70513 (12)	0.0540 (7)	
F4	0.30581 (18)	0.7775 (2)	0.63902 (15)	0.0586 (7)	
F5	0.3608 (2)	0.7641 (3)	0.74431 (16)	0.0840 (11)	
F6	0.4135 (2)	0.9157 (2)	0.62167 (13)	0.0588 (7)	
N1	0.40722 (19)	0.5353 (2)	0.58640 (13)	0.0231 (6)	
H1	0.424 (3)	0.5969 (18)	0.6075 (15)	0.027 (9)*	
N2	0.48361 (19)	0.3368 (2)	0.53888 (14)	0.0248 (6)	
N3	0.4460 (2)	0.2326 (2)	0.62505 (14)	0.0282 (6)	
N4	0.3548 (2)	0.4794 (2)	0.44007 (14)	0.0267 (6)	
C1	0.4281 (3)	0.4463 (3)	0.63589 (16)	0.0274 (7)	
H1A	0.3690	0.4358	0.6648	0.033*	
H1B	0.4861	0.4685	0.6635	0.033*	
C2	0.4510 (2)	0.3384 (3)	0.60004 (16)	0.0243 (7)	
C3	0.4784 (3)	0.1600 (3)	0.57608 (19)	0.0326 (8)	
H3	0.4838	0.0800	0.5790	0.039*	
C4	0.5009 (2)	0.2245 (3)	0.5236 (2)	0.0294 (7)	
H4	0.5248	0.1972	0.4825	0.035*	
C5	0.4136 (3)	0.1995 (3)	0.69075 (18)	0.0372 (9)	
H5A	0.3915	0.2667	0.7150	0.056*	
H5B	0.4697	0.1638	0.7141	0.056*	
H5C	0.3578	0.1457	0.6874	0.056*	
C6	0.2980 (2)	0.5415 (3)	0.57167 (17)	0.0254 (7)	
H6A	0.2621	0.5715	0.6106	0.031*	
H6B	0.2724	0.4643	0.5629	0.031*	
C7	0.2758 (2)	0.6170 (3)	0.51236 (17)	0.0292 (7)	
H7A	0.2092	0.6529	0.5181	0.035*	
H7B	0.3267	0.6778	0.5099	0.035*	
C8	0.2766 (2)	0.5506 (3)	0.44925 (17)	0.0277 (7)	
C9	0.1977 (3)	0.5560 (4)	0.40485 (19)	0.0391 (9)	
H9	0.1448	0.6087	0.4116	0.047*	
C10	0.1957 (3)	0.4854 (4)	0.3511 (2)	0.0498 (11)	
H10	0.1425	0.4895	0.3201	0.060*	
C11	0.2727 (3)	0.4086 (4)	0.34328 (19)	0.0449 (10)	
H11	0.2724	0.3564	0.3078	0.054*	
C12	0.3505 (3)	0.4095 (3)	0.38849 (17)	0.0330 (8)	
H12	0.4040	0.3573	0.3825	0.040*	

### Atomic displacement parameters (Å^2^)

**Table:**

	*U*^11^	*U*^22^	*U*^33^	*U*^12^	*U*^13^	*U*^23^
Zn1	0.0169 (3)	0.0213 (3)	0.0247 (3)	−0.00221 (18)	0.00329 (19)	0.00056 (19)
P1	0.0221 (4)	0.0233 (4)	0.0299 (5)	0.0017 (3)	−0.0017 (3)	−0.0007 (3)
F1	0.0394 (12)	0.0431 (13)	0.0747 (18)	0.0150 (11)	−0.0061 (12)	−0.0213 (12)
F2	0.0428 (13)	0.0550 (15)	0.0710 (18)	0.0110 (12)	−0.0256 (12)	−0.0275 (13)
F3	0.0449 (13)	0.0636 (16)	0.0534 (15)	0.0283 (12)	−0.0118 (11)	−0.0196 (12)
F4	0.0410 (13)	0.0396 (13)	0.095 (2)	−0.0040 (10)	−0.0220 (13)	−0.0184 (13)
F5	0.0588 (18)	0.106 (3)	0.087 (2)	0.0105 (17)	0.0142 (15)	0.069 (2)
F6	0.0594 (16)	0.0596 (16)	0.0573 (17)	−0.0066 (13)	−0.0012 (13)	0.0243 (13)
N1	0.0209 (12)	0.0230 (12)	0.0253 (15)	−0.0035 (10)	0.0010 (10)	−0.0006 (11)
N2	0.0218 (13)	0.0216 (12)	0.0309 (16)	−0.0020 (10)	0.0017 (11)	0.0006 (11)
N3	0.0248 (13)	0.0270 (14)	0.0328 (16)	−0.0074 (11)	−0.0075 (11)	0.0064 (12)
N4	0.0197 (13)	0.0328 (14)	0.0277 (15)	−0.0047 (11)	0.0012 (11)	0.0005 (11)
C1	0.0271 (15)	0.0277 (16)	0.0273 (18)	−0.0017 (13)	0.0014 (13)	0.0013 (13)
C2	0.0196 (14)	0.0218 (15)	0.0317 (18)	−0.0034 (12)	−0.0024 (12)	0.0040 (13)
C3	0.0272 (16)	0.0223 (15)	0.048 (2)	−0.0028 (13)	−0.0097 (15)	0.0008 (15)
C4	0.0221 (15)	0.0250 (16)	0.041 (2)	−0.0027 (12)	−0.0029 (14)	−0.0050 (14)
C5	0.043 (2)	0.0341 (18)	0.035 (2)	−0.0167 (16)	−0.0106 (16)	0.0121 (15)
C6	0.0171 (14)	0.0282 (15)	0.0310 (18)	−0.0024 (12)	0.0061 (12)	−0.0010 (13)
C7	0.0164 (14)	0.0285 (16)	0.043 (2)	0.0007 (12)	0.0027 (13)	0.0011 (14)
C8	0.0176 (14)	0.0335 (17)	0.0321 (19)	−0.0035 (12)	0.0036 (13)	0.0048 (14)
C9	0.0227 (17)	0.057 (2)	0.037 (2)	0.0016 (16)	−0.0007 (15)	0.0107 (18)
C10	0.0278 (19)	0.087 (3)	0.034 (2)	−0.006 (2)	−0.0050 (16)	0.003 (2)
C11	0.0316 (19)	0.076 (3)	0.027 (2)	−0.012 (2)	0.0021 (15)	−0.0128 (19)
C12	0.0235 (16)	0.044 (2)	0.031 (2)	−0.0061 (15)	0.0052 (13)	−0.0049 (15)

### Geometric parameters (Å, °)

**Table:**

Zn1—N1	2.188 (3)	C1—H1A	0.9900
Zn1—N1^i^	2.188 (3)	C1—H1B	0.9900
Zn1—N2^i^	2.095 (3)	C3—C4	1.345 (5)
Zn1—N2	2.095 (3)	C3—H3	0.9500
Zn1—N4	2.298 (3)	C4—H4	0.9500
Zn1—N4^i^	2.298 (3)	C5—H5A	0.9800
P1—F5	1.549 (3)	C5—H5B	0.9800
P1—F2	1.583 (2)	C5—H5C	0.9800
P1—F3	1.588 (2)	C6—C7	1.529 (5)
P1—F4	1.595 (2)	C6—H6A	0.9900
P1—F6	1.601 (3)	C6—H6B	0.9900
P1—F1	1.605 (2)	C7—C8	1.504 (5)
N1—C1	1.482 (4)	C7—H7A	0.9900
N1—C6	1.486 (4)	C7—H7B	0.9900
N1—H1	0.875 (10)	C8—C9	1.386 (5)
N2—C2	1.317 (4)	C9—C10	1.375 (6)
N2—C4	1.382 (4)	C9—H9	0.9500
N3—C2	1.351 (4)	C10—C11	1.378 (6)
N3—C3	1.383 (5)	C10—H10	0.9500
N3—C5	1.457 (4)	C11—C12	1.385 (5)
N4—C12	1.336 (4)	C11—H11	0.9500
N4—C8	1.352 (4)	C12—H12	0.9500
C1—C2	1.500 (4)		
			
N2^i^—Zn1—N2	180.00 (6)	C2—C1—H1A	110.1
N2^i^—Zn1—N1	100.73 (10)	N1—C1—H1B	110.1
N2—Zn1—N1	79.27 (10)	C2—C1—H1B	110.1
N2^i^—Zn1—N1^i^	79.27 (10)	H1A—C1—H1B	108.4
N2—Zn1—N1^i^	100.73 (10)	N2—C2—N3	111.0 (3)
N1—Zn1—N1^i^	180.0	N2—C2—C1	122.5 (3)
N2^i^—Zn1—N4	89.13 (10)	N3—C2—C1	126.4 (3)
N2—Zn1—N4	90.87 (10)	C4—C3—N3	106.8 (3)
N1—Zn1—N4	88.35 (10)	C4—C3—H3	126.6
N1^i^—Zn1—N4	91.65 (9)	N3—C3—H3	126.6
N2^i^—Zn1—N4^i^	90.87 (10)	C3—C4—N2	109.2 (3)
N2—Zn1—N4^i^	89.13 (10)	C3—C4—H4	125.4
N1—Zn1—N4^i^	91.65 (9)	N2—C4—H4	125.4
N1^i^—Zn1—N4^i^	88.35 (10)	N3—C5—H5A	109.5
N4—Zn1—N4^i^	180.0	N3—C5—H5B	109.5
F5—P1—F2	91.12 (18)	H5A—C5—H5B	109.5
F5—P1—F3	91.64 (17)	N3—C5—H5C	109.5
F2—P1—F3	91.04 (13)	H5A—C5—H5C	109.5
F5—P1—F4	92.36 (18)	H5B—C5—H5C	109.5
F2—P1—F4	176.28 (17)	N1—C6—C7	112.2 (2)
F3—P1—F4	90.16 (13)	N1—C6—H6A	109.2
F5—P1—F6	179.05 (19)	C7—C6—H6A	109.2
F2—P1—F6	89.42 (16)	N1—C6—H6B	109.2
F3—P1—F6	89.12 (15)	C7—C6—H6B	109.2
F4—P1—F6	87.08 (15)	H6A—C6—H6B	107.9
F5—P1—F1	90.41 (17)	C8—C7—C6	111.6 (3)
F2—P1—F1	88.66 (13)	C8—C7—H7A	109.3
F3—P1—F1	177.93 (16)	C6—C7—H7A	109.3
F4—P1—F1	90.02 (13)	C8—C7—H7B	109.3
F6—P1—F1	88.83 (15)	C6—C7—H7B	109.3
C1—N1—C6	110.8 (2)	H7A—C7—H7B	108.0
C1—N1—Zn1	107.68 (19)	N4—C8—C9	121.5 (3)
C6—N1—Zn1	113.6 (2)	N4—C8—C7	116.6 (3)
C1—N1—H1	102 (2)	C9—C8—C7	121.8 (3)
C6—N1—H1	108 (2)	C10—C9—C8	120.3 (4)
Zn1—N1—H1	114 (2)	C10—C9—H9	119.8
C2—N2—C4	106.3 (3)	C8—C9—H9	119.8
C2—N2—Zn1	112.2 (2)	C9—C10—C11	118.5 (4)
C4—N2—Zn1	141.4 (2)	C9—C10—H10	120.8
C2—N3—C3	106.7 (3)	C11—C10—H10	120.8
C2—N3—C5	127.4 (3)	C10—C11—C12	118.3 (4)
C3—N3—C5	125.9 (3)	C10—C11—H11	120.8
C12—N4—C8	117.4 (3)	C12—C11—H11	120.8
C12—N4—Zn1	121.2 (2)	N4—C12—C11	123.9 (4)
C8—N4—Zn1	120.6 (2)	N4—C12—H12	118.0
N1—C1—C2	108.1 (3)	C11—C12—H12	118.0
N1—C1—H1A	110.1		
			
N2^i^—Zn1—N1—C1	−152.89 (19)	Zn1—N2—C2—C1	0.5 (4)
N2—Zn1—N1—C1	27.11 (19)	C3—N3—C2—N2	0.4 (4)
N4—Zn1—N1—C1	118.3 (2)	C5—N3—C2—N2	−180.0 (3)
N4^i^—Zn1—N1—C1	−61.7 (2)	C3—N3—C2—C1	−176.9 (3)
N2^i^—Zn1—N1—C6	84.0 (2)	C5—N3—C2—C1	2.7 (5)
N2—Zn1—N1—C6	−96.0 (2)	N1—C1—C2—N2	23.2 (4)
N4—Zn1—N1—C6	−4.8 (2)	N1—C1—C2—N3	−159.8 (3)
N4^i^—Zn1—N1—C6	175.2 (2)	C2—N3—C3—C4	−0.4 (4)
N1—Zn1—N2—C2	−15.5 (2)	C5—N3—C3—C4	179.9 (3)
N1^i^—Zn1—N2—C2	164.5 (2)	N3—C3—C4—N2	0.3 (4)
N4—Zn1—N2—C2	−103.7 (2)	C2—N2—C4—C3	−0.1 (4)
N4^i^—Zn1—N2—C2	76.3 (2)	Zn1—N2—C4—C3	175.0 (3)
N1—Zn1—N2—C4	169.6 (4)	C1—N1—C6—C7	−169.8 (3)
N1^i^—Zn1—N2—C4	−10.4 (4)	Zn1—N1—C6—C7	−48.4 (3)
N4—Zn1—N2—C4	81.4 (4)	N1—C6—C7—C8	90.1 (3)
N4^i^—Zn1—N2—C4	−98.6 (4)	C12—N4—C8—C9	−3.8 (5)
N2^i^—Zn1—N4—C12	109.5 (3)	Zn1—N4—C8—C9	165.9 (3)
N2—Zn1—N4—C12	−70.5 (3)	C12—N4—C8—C7	171.8 (3)
N1—Zn1—N4—C12	−149.8 (3)	Zn1—N4—C8—C7	−18.5 (4)
N1^i^—Zn1—N4—C12	30.2 (3)	C6—C7—C8—N4	−47.0 (4)
N2^i^—Zn1—N4—C8	−59.9 (2)	C6—C7—C8—C9	128.7 (3)
N2—Zn1—N4—C8	120.1 (2)	N4—C8—C9—C10	2.3 (6)
N1—Zn1—N4—C8	40.9 (2)	C7—C8—C9—C10	−173.1 (3)
N1^i^—Zn1—N4—C8	−139.1 (2)	C8—C9—C10—C11	1.0 (6)
C6—N1—C1—C2	92.0 (3)	C9—C10—C11—C12	−2.6 (6)
Zn1—N1—C1—C2	−32.7 (3)	C8—N4—C12—C11	2.2 (5)
C4—N2—C2—N3	−0.2 (3)	Zn1—N4—C12—C11	−167.5 (3)
Zn1—N2—C2—N3	−176.9 (2)	C10—C11—C12—N4	1.1 (6)
C4—N2—C2—C1	177.2 (3)		

Symmetry codes: (i) −*x*+1, −*y*+1, −*z*+1.

### Hydrogen-bond geometry (Å, °)

**Table:**

*D*—H···*A*	*D*—H	H···*A*	*D*···*A*	*D*—H···*A*
N1—H1···F1	0.88 (1)	2.21 (1)	3.083 (4)	179 (3)

## References

[bb1] Barbour, L. J. (2001). *J. Supramol. Chem.* **1**, 189–191.

[bb2] Bruker (2005). *APEX2* and *SAINT* Bruker AXS Inc., Madison, Wisconsin, USA.

[bb3] Greatti, A., Scarpellini, M., Peralta, R. A., Bortoluzi, A. J., Xavier, F. R., Szoganicz, B., Tomkowicz, Z., Rams, M., Haase, W. & Neves, A. (2008). *Inorg. Chem.* **47**, 1107–1119. 10.1021/ic702132t 18181617

[bb4] Sheldrick, G. M. (1996). *SADABS* University of Göttingen, Germany.

[bb5] Sheldrick, G. M. (2008). *Acta Cryst.* A**64**, 112–122. 10.1107/S0108767307043930 18156677

[bb6] Westrip, S. P. (2009). *publCIF* In preparation.

